# An Unexplored Pharmacologic/Diagnostic Strategy for Peri-Implantitis: A Protocol Proposal

**DOI:** 10.3390/diagnostics10121050

**Published:** 2020-12-05

**Authors:** Lorne M. Golub, Ismo T. Räisänen, Timo Sorsa, Philip M. Preshaw

**Affiliations:** 1 Department of Oral Biology and Pathology, School of Dental Medicine, Health Sciences Center, Stony Brook University, Stony Brook, NY 11794, USA; Lorne.Golub@stonybrookmedicine.edu; 2 Department of Oral and Maxillofacial Diseases, Head and Neck Center, University of Helsinki and Helsinki University Hospital, PO Box 63 (Haartmaninkatu 8), FI-00014 Helsinki, Finland; timo.sorsa@helsinki.fi; 3 Division of Periodontology, Department of Dental Medicine, Karolinska Institutet, SE-171 77 Stockholm, Sweden; 4 National University Centre for Oral Health, Faculty of Dentistry, National University of Singapore, Singapore 119077, Singapore; philip.preshaw@nus.edu.sg; 5 School of Dental Sciences, Newcastle University, Newcastle upon Tyne NE1 7RU, UK

**Keywords:** dental implants, peri-implant mucositis, peri-implantitis, periodontitis, host modulation therapies, matrix metalloproteinases, matrix metalloproteinase 8, aMMP-8

## Abstract

Dental implants are widely utilized for the replacement of missing teeth and are increasingly being placed in patients with systemic diseases, as well as in those who are medically healthy. Furthermore, it is recognized that peri-implant mucositis and peri-implantitis are highly prevalent, affecting large numbers of patients with implants, and it is pertinent to consider whether there may be any systemic impact of these conditions, given that there are known links between periodontitis and a number of chronic inflammatory diseases. In this article, we propose that the potential systemic complications of peri-implant diseases should be investigated in future clinical research, together with studies to identify whether systemically-administered host modulation therapies (HMTs) may be of benefit in the treatment of peri-implant diseases. These “HMTs” may prove a useful adjunct to routinely employed debridement and disinfection protocols, as well as potentially being of benefit in reducing risks of systemic complications. We also consider the use of chair-side diagnostic tests for active matrix metalloproteinase-8 (aMMP-8) in the detection of peri-implant disease given the ability of such tests to detect active tissue breakdown associated with peri-implantitis and periodontitis before conventional clinical and radiographic measurements indicate pathologic changes. These novel diagnostic and therapeutic strategies are relevant to consider as they may improve the management of peri-implant disease (beyond local debridement procedures), especially in those patients in whom systemic inflammation might be of concern.

Dental implants have become a dominant treatment strategy to enhance oral function and esthetics in partially and completely edentulous patients. Increasingly, however, implants are being placed not only in medically healthy individuals, but also in those with systemic disorders such as diabetes, cardiovascular and gastrointestinal disease [1,2,3]. Although many case reports of implant placement in medically compromised patients have been described as successful, it has also been stated that, currently, “there is insufficient evidence to determine whether dental implants can remain functionally stable” over prolonged periods of time in systemically compromised patients [1] and this is an area of ongoing research.

While the issue of local (oral) inflammation in dental implantology has been extensively addressed in a number of studies (see Javed and Romanos [1] for a review), we now consider that an additional question should be posed; that is, what is the risk, if any, that peri-implant disease may induce and/or contribute to systemic inflammation? This question is relevant because, firstly, the prevalence rates of peri-implant mucositis and peri-implantitis are high [4]; it has been estimated that peri-implant mucositis affects about 43%, and peri-implantitis 22%, of patients with implants [5]. Secondly, the inflammatory response in peri-implantitis appears to produce larger [6], and more intense and tissue-destructive lesions than that in periodontitis, with bone loss occurring in a non-linear, accelerating pattern [7]. Regarding mechanistic support of this enhanced pathology, it has been reported that peri-implant sulcular fluid (PISF) contains higher levels of leukocyte-type collagenase (matrix metalloproteinase-8, MMP-8) than gingival crevicular fluid (GCF) around natural teeth [8]. Furthermore, peri-implant sites often have much larger inflammatory lesions with significantly higher proportions, numbers and densities of cells part of both the innate and the adaptive host response, namely the plasma cells, macrophages and neutrophils compared with periodontitis sites [6]. Furthermore, higher numbers of M1 tissue-destructive macrophages (rather than M2 anti-inflammatory macrophages) have been found to infiltrate peri-implantitis lesions compared to those with periodontitis [9]. Moreover, it is well known that systemic inflammation is associated with increased risk of cardiovascular and other systemic diseases such as diabetes [5,10,11]. It is also clear that diabetes and smoking, both of which have impacts on systemic inflammation, are key risk factors for periodontal and peri-implant diseases. Thus, upregulated systemic (as well as local) inflammation likely underpins the links between periodontitis (and possibly peri-implantitis, though this has yet to be demonstrated) and diabetes and cardiovascular diseases. 

Accordingly, we now propose that the potential systemic complications of peri-implant diseases should be considered by the profession and investigated in appropriately designed clinical trials. If such an impact is confirmed, this would suggest the need for more intensive treatment of peri-implantitis, which would include not only commonly employed local debridement and disinfection protocols, but also might incorporate currently available (and future) systemically-administered (oral route) host modulation therapies (HMTs); those currently available were reviewed recently and are briefly discussed below [10]. This proposed “combination therapy” may not only enhance the efficacy of conventional disinfection treatment protocols for peri-implantitis but may also reduce the risks of any systemic complications as well.

Thus, we suggest that in future studies of peri-implantitis, patients should be analyzed for well-established biomarkers of systemic inflammation (for example, high sensitivity C-reactive protein (hsCRP), cytokines such as interleukin (IL)-6, and collagenolytic enzymes such as MMP-8, MMP-9) in their blood samples, before and after local debridement procedures, and in combination with HMT. Evidence to support the potential benefits of this approach has already been demonstrated. In an National Institutes of Health (NIH)-sponsored, double-blind, placebo-controlled study of 128 post-menopausal women, who exhibited both local (periodontitis) and systemic (osteopenia) bone loss, a 2 year regimen of this proposed therapeutic strategy significantly reduced biomarkers of systemic inflammation (hsCRP and MMP-9 in their blood samples), as well as reducing local (GCF) and systemic (blood) levels of collagenolytic enzymes (MMP-8) and bone resorption biomarkers (ICTP, carboxy-terminal telopeptide fragments of type I collagen) [10,12,13,14]. In an earlier double-blind placebo-controlled clinical trial of patients with severe cardiovascular disease (acute coronary syndrome, ACS), a similar HMT (non-antibiotic-dose doxycycline) demonstrated efficacy in reducing biomarkers of systemic inflammation after a 6 month regimen [15,16]. Moreover, the clinical importance of this proposed strategy is based on numerous clinical and epidemiologic studies in cardiology that have consistently demonstrated that systemic inflammation (characterized by elevated levels of hsCRP, cytokines, and MMPs in the circulation) is a greater risk for myocardial infarction than elevated cholesterol [17,18].

An additional innovation to consider is the development of diagnostic chair-side tests for active MMP-8 (aMMP-8), the presence of which indicates active periodontal tissue breakdown. Historically, this was suggested in 1974–1976 in a series of articles that described the detection and “characteristics of collagenase activity in gingival crevicular fluid and its relationship to gingival diseases” (i.e., periodontitis, pericoronitis) in humans [19,20,21,22]. Since that time, a number of potential targets have been investigated for utility in the detection and monitoring of periodontitis, such as MMP-8, MMP-13, MMP-14, myeloperoxidase and azurocidin [23]. These studies have consistently identified MMP-8 as a most promising candidate for oral fluid (GCF, PISF, mouthrinse, saliva) point-of-care diagnostics and for evaluating disease progression and treatment outcomes [24]. In parallel, a number of both qualitative and quantitative technologies for analysis of MMP-8 levels in oral samples have been developed, including immunofluorometric assays (IFMA), an MMP-8 specific immunochromatographic dip-stick test, sandwich-based immunoassay systems (MMP-8 DentoAnalyzer device), and conventional MMP-8 ELISAs (enzyme-linked immunosorbent assays) [25]. 

Given the importance of MMP-8, and more specifically aMMP-8 in the pathogenesis of periodontal diseases, more recently, chair-side tests (government-approved in Europe) have been developed that can measure aMMP-8 levels in PISF, GCF and mouthrinse, and have been validated in studies conducted in the USA, Africa, and Europe [26,27,28,29,30,31]. Its advantage, beyond traditional diagnostic procedures (e.g., bleeding on probing, pocket depth), is enhanced diagnostic sensitivity and specificity, including the ability to predictively detect peri-implantitis and periodontitis before clinical and radiographic measurements indicate pathologic changes [26,27,28,29,30,31]. Further studies are required to investigate the utility of this test in different patient populations, such as smokers and non-smokers, given that GCF and serum MMP-8 levels are known to vary between smokers and non-smokers [24,32]. It is also necessary to develop reproducible reference ranges, for example to distinguish between health, gingivitis, or periodontitis, but perhaps more usefully to identify (with adequate sensitivity and specificity) disease progression, or periodontal stability following successful treatment. 

Recently, Sorsa et al. [33] suggested reference ranges for aMMP-8 in mouthrinse (measured by PerioSafe® chair-side/point-of-care aMMP-8 test [29,33]) in regard to grading a periodontitis patient [34]. Similarly, aMMP-8 reference ranges can be proposed for grading peri-implantitis patients and their risk of disease progression. Based on a recent study by Lähteenmäki et al. [31], a cut-off of 20 ng/mL for aMMP-8 levels measured in PISF by ImplantSafe® chair-side/point-of-care aMMP-8 test identified all 26 peri-implantitis patients and 26 healthy dental implant controls correctly. Furthermore, the aMMP-8 chair-side test was more accurate than other potential biomarkers such as neutrophil elastase, myeloperoxidase, pro and active forms of MMP-9 and bleeding on probing [31]. With the same study group, we have investigated this further and found that the aMMP-8 chair-side test was also more accurate than calprotectin and gelatinases (25–50 kDa, 95–150 kDa, over 150 kDa and total gelatinolytic activity) (see Figure 1). Thus, based on the current evidence, grading the risk of periodontal and peri-implant disease progression by utilizing aMMP-8 can be summarized: low aMMP-8 levels are associated with periodontal and peri-implant health and low risk of disease progression, while elevated levels indicate elevated risk of periodontitis and peri-implantitis and their progression (Table 1) [26,27,28,29,30,31,33]. It should be noted that the ImplantSafe® and PerioSafe® lateral flow immunotests utilize the same antibody for aMMP-8 measurements [25,29,33], because different antibodies with differing sensitivities and specificities to target MMP-8 isotypes can give significantly different aMMP-8 results. For example, previously, Sorsa et al. [25] and Nieminen et al. [35] found a strong correlation between IFMA and ELISA assays for aMMP-8 measurements with this same antibody [25,29,33], but not with Amersham ELISA kit, which utilizes a different antibody.

Clearly, much research is yet to be undertaken to evaluate the potential use of diagnostic chair-side tests in identifying the onset and progression of peri-implant diseases. Peri-implantitis is a complex chronic inflammatory condition, in which multiple systemic (e.g., smoking, diabetes), behavioral (e.g., patient compliance with oral hygiene and maintenance visits), local (e.g., presence of periodontitis) and prosthetic (e.g., excess cement, poorly fitting prostheses, micro gaps at the implant–abutment interface) all contribute to the risk of developing the disease [36,37]. We also recognize that a beneficial impact of the novel therapeutic and diagnostic strategies, described above, in reducing local and systemic inflammation in patients with periodontitis (which often affects multiple teeth), might not produce a detectable similar systemic response in patients with peri-implantitis, since these individuals typically have fewer numbers of implant placements. However, such studies are likely to advance our profession’s management of peri-implant disease (beyond local debridement procedures), especially in those patients in whom systemic inflammation might be of concern. Related to the dominance of innate immune responses and defense responses in peri-implantitis instead of bacterial response systems [38], HMTs, as an adjunct to debridement procedures (and combined with real-time PISF diagnostics), may provide an effective and comprehensive therapeutic strategy. The more intense inflammatory character of peri-implantitis lesions pose an elevated risk of rapid progression that underscores the importance of timely and effective disease diagnostics and treatment [39].

## Figures and Tables

**Figure 1:**
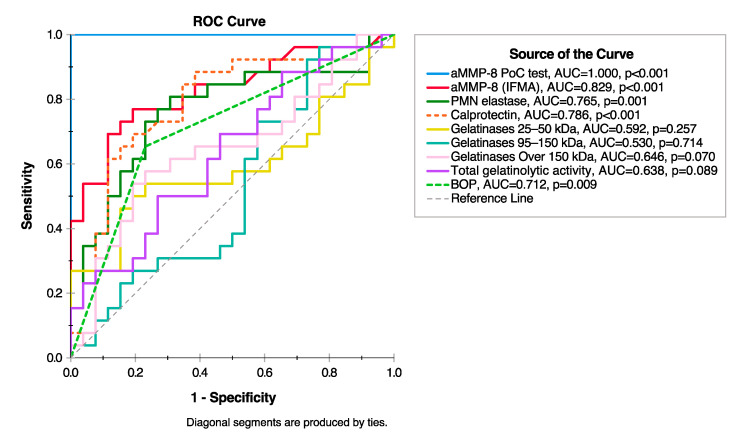
Receiver operating characteristic (ROC) analysis illustrating the diagnostic ability of active matrix metalloproteinase-8 (aMMP-8) chair-side/point-of-care (PoC) test and selected potential biomarkers: polymorphonuclear leukocyte (PMN)/neutrophil elastase, calprotectin, gelatinases (25–50 kDa, 95–150 kDa, over 150 kDa and total gelatinolytic activity) and bleeding on probing (BOP) to discriminate peri-implantitis from healthy dental implant (26 peri-implant and 26 healthy dental implant patients). aMMP-8 PoC test and selected potential biomarkers were assessed as described previously [31]. This figure has been constructed by authors ITR and TS based on the same study group as described in Lähteenmäki et al. and further extends their results [31].

**Table 1:** Grading of peri-implantitis and periodontitis and the risk of their progression (modified from Tonetti et al. [34] and Sorsa et al. [33]) by active matrix metalloproteinase-8 (aMMP-8) as the main biomarker for active/progressing peri-implant and periodontal diseases. Here, the aMMP-8 cut-offs for peri-implantitis and periodontitis are applicable to ImplantSafe® and PerioSafe® lateral flow aMMP-8 point-of-care immunotests utilizing the same antibody in the aMMP-8 measurements [25,29,33].

Grading of the Risk of Disease Progression in Peri-Implantitis and Periodontitis by aMMP-8			
		No or Low Risk	Elevated Risk
Indicators of active periodontal tissue destruction/bone loss/clinical attachment loss	Peri-implant sulcular fluid, gingival crevicular fluid, mouthrinse	aMMP-8 concentration below 20 ng/mL	aMMP-8 concentration ≥20 ng/mL
